# Changes of Lacrimal Puncta by Anterior Segment Optical Coherence Tomography after Topical Combined Antibiotic and Steroid Treatment in Cases of Inflammatory Punctual Stenosis

**DOI:** 10.1155/2022/7988091

**Published:** 2022-01-24

**Authors:** Islam Awny, Elshimaa A. Mateen Mossa, Tasneem Mohammed Bakheet, Hany Mahmoud, Amr Mounir

**Affiliations:** ^1^Sohag Faculty of Medicine, Ophthalmology Department, Sohag University, Sohag, Egypt; ^2^Sohag Faculty of Medicine, Public Health and Community Medicine Department, Sohag University, Sohag, Egypt

## Abstract

**Purpose:**

To evaluate the role of medical treatment and assessing its effect on resolving epiphora and improving punctum size by high resolution AS-OCT imaging comparing punctal parameters in patients before and after treatment with topical combined antibiotic and steroid treatment in cases of inflammatory punctual stenosis. *Patients and Methods*. Double-blinded controlled randomized study which was conducted on two groups of patients who had acquired punctal stenosis and epiphora presented to Ophthalmology Clinics of Sohag University Hospitals in the period between Jan 2021 and April 2021. The study included 44 eyes of 50 subjects complaining of epiphora. They were divided into two groups, the epiphora group one (EG1) received eye drops containing combination of antibiotics and steroids (orchadexoline eye drops, each ml contains 5 mg chloramphenicol, 1 mg dexamethasone sodium phosphate, 0.25 mg tetryzoline hydrochloride, 2 mg hydroxypropyl methyl cellulose, 10 mg *α*-tocopherol acetate (vitamin E), and 8 mg macrogol 400), 5 times daily for the first week, three times daily for the next two weeks, and one time daily for another one week. The second epiphora group (EG2) received preservative-free artificial tears (sodium hyaluronate-, polyethylene-, and propylene glycol-based), three times daily for four weeks. The patients were examined before treatment, one week, one month, and one and half months later.

**Results:**

Both groups were comparable regarding mean age (49 ± 13 vs 53 ± 11 years, *P* value = 0.2) and sex (males were 38.6% vs 31.8%, female were 61.4% vs 68.2%, *P* value = 0.6), respectively, with no statistically significant difference between both groups. Both groups were comparable regarding outer punctual diameter and length between the puncti before treatment. Outer punctal diameters were (EG1 228 ± 113 um, EG2 241 ± 115 um, *P* value = 0.5). Length between the puncti were (EG1 129 ± 73 um, EG2 137 ± 72 um, *P* value = 0.6). There was marked improvement of the outer punctual diameter (EG1 373 um ± 92 um, EG2 240 ± 109 um, (*P* value < 0.0001) and length between the puncti (EG1 217 ± 109 um, 136 ± 71 um (*P* value < 0.0002)) during the follow-up period. EG1 showed more improvement than EG2 when compared during the follow-up period.

**Conclusions:**

Topical combined antibiotic and steroid treatment was an effective method in treating cases of inflammatory punctual stenosis as found by monitoring of punctal parameter changes by AS-OCT. AS-OCT was found to be a useful method for evaluation of the lacrimal punctal parameters especially with different treatment modalities in epiphora cases.

## 1. Introduction

Lacrimal punctal stenosis is one of the least-evaluated etiologies of epiphora [1]. It is defined as narrowing or occlusion of the external opening of the lacrimal canaliculi, while the distal tear drainage system is free [2, 3]. It can also be defined more precisely as the punctum size less than 0.3 mm or inability to cannulate it with a 26G cannula without dilation [4].

Acquired punctal stenosis can result from variable causes, of which eye trauma, local or systemic drug toxicity, inflammatory or infectious diseases, malposition of the eyelid, tumors of upper and lower lid, or even the ageing changes could lead to that [5].

Various noxious stimuli can cause chronic inflammation with subsequent fibrosis and stenosis [6]. Punctal stenosis can be associated with canalicular, nasolacrimal sac and duct stenosis or obstruction in some cases [7].

Several methods were used for measuring normal punctum parameters, of which fitting different gauge cannulae (20–32G) but stretchable punctal walls during intubation made this method an unreliable predictor of punctal parameter measurements under normal physiological conditions [3].

Other methods approved photographing all four puncta by slit-lamp biomicroscopy and by using computer cursor punctal borders which are later mapped out, and the punctal area is measured by software-driven computer analysis [8].

Others used Ramsden eyepiece which has of a fixed transparent graduated scale positioned on the field and fitted to the slit-lamp with a fixed optical magnification of 32x results in a measurement of 0.03 mm [9].

AS-OCT parameters were (614.6 ± 195.6 um) for the outer punctal diameter and (545.8 ± 270.1 um) for the punctal depth. [10].

Anterior segment optical coherence tomography (AS-OCT) is accurate, noninvasive, objective, safe, and cross-sectional imaging method used to study various parts of the anterior segment of the eye including cornea, conjunctiva, angle, the lower tear meniscus height, and the lacrimal punctal parameters [10, 11].

In the literature, several methods were used for grading punctal stenosis. One of the most commonly used methods is Kashkouli's scoring system (grade 0 no punctum (agenesis), grade 1 punctal papilla is covered with a membrane (difficult to recognize), and the grade is less than the normal size but recognizable, grade 3 punctum is normal, grade 4 punctum is small slit (<2 mm), and grade 5 punctum is large slit (≤2 mm)) [12].

Munk scale for epiphora grading is another grading system (grade 0 no epiphora, grade 1 epiphora requiring dabbing less than twice a day, grade 2 epiphora requiring dabbing 2–4 times a day, grade 3 epiphora requiring dabbing 5–10 times a day, grade 4 epiphora requiring dabbing more than 10 times a day, and grade 5 is constant epiphora) [13].

The aim of this work was to evaluate the role of medical treatment and assessing its effect on resolving epiphora and improving punctum size by high resolution AS-OCT imaging comparing punctal parameters of the patients before and after treatment with topical combined antibiotic and steroids treatment in cases of inflammatory punctual stenosis.

## 2. Patients and Methods

The study included 44 eyes of 50 subjects; total coverage of epiphora patients fulfilled inclusion criteria and presented to the ophthalmology clinics of Sohag University Hospitals in the period between Jan 2021 and April 2021.

Patients included were aged 21 years or more who developed acquired inflammatory punctal edema and were complaining of epiphora. Punctal stenosis grading of the included groups was grade 2 according to Kashkouli's classification [12], and epiphora grading was 1 : 5 according to Munk's classification [13].

Patients who had a history of previous ocular and lacrimal surgery, trauma, lower lid margin malposition or laxity, dry eye (for the control group), glaucoma, corneal abnormalities (abrasions and keratitis), congenital punctal anomalies, nasolacrimal duct obstruction, mucocele, and pyocele were excluded.

The study design is a double-blinded controlled randomized study which was conducted on two group of patients, the first group, who had acquired punctal stenosis and epiphora (EG1) who received eye drops contains combination of antibiotics and steroids (orchadexoline eye drops, each ml contains 5 mg chloramphenicol, 1 mg dexamethasone sodium phosphate, 0.25 mg tetryzoline hydrochloride, 2 mg hydroxypropyl methyl cellulose, 10 mg *α*-tocopherol acetate (vitamin E), and 8 mg macrogol 400). The patients were advised to apply the drops 5 times daily for the first week, three times daily for the next two weeks, and one time daily for another one week. The second epiphora group (EG2) who received preservative-free artificial tears (sodium hyaluronate-, polyethylene-, and propylene glycol-based) three times daily for four weeks.

All patients were examined before treatment, one week, one month, and one and half months later. Orchadexoline eye drops were chosen due to its characteristic bitter taste to have a subjective method for evaluation of relieving of stenosis in addition to the objective method by AS-OCT. As the patients said that they felt bitter taste during treatment which is considered a sign of improvement.

Patients' selection was based on slit-lamp examination of the eye lids, corneal surface, bulbar and palpebral conjunctiva, tear meniscus, intraocular pressure (IOP) measurement, lower punctal examination, and grading of both punctal stenosis and epiphora according to Kashkouli's and Munk scoring systems [12].

### 2.1. Spectral Domain Anterior Segment-OCT Image Acquisition

AS-OCT for lower puncti was performed using RTVue (Optovue Inc., Fermont, CA). OCT images of the width and length of the lower puncti of the participants were captured by the same operator on the same machine. The lower eyelid margin was gently everted using a cotton bud that was placed below the punctum. The punctum was everted perpendicular to the light source to allow alignment of the punctum depth with respect to the axis of the scanner's infrared beam. The punctum was imaged with the scan line placed horizontally along the mucocutaneous junction. Outer punctal diameter was measured as the distance between the two highest points on the nasal and temporal punctal orifice. Punctal depth is the vertical lumen; it was measured vertically between outer and inner punctal openings which appears closed.

This study was approved by the Ethical Committee of Faculty of Medicine, Sohag University, under IBR registration number S20-159 with clinical trial registration number NCT05028907 and was performed according to the guidelines and Declaration of Helsinki. The aim of the study and intervention details were discussed with the participants, and informed written consent was obtained from them before inclusion.

### 2.2. Statistical Analysis

Statistical analysis was conducted using the SPSS software (version 26.0) (SPSS Inc., Chicago, IL, USA). All participants were chosen by using the systematic random sample technique from the attendees who fulfilled the inclusion criteria. The data were tested for normality using the Kolmogorov–Smirnov test and for homogeneity variances before further statistical analysis. Differences between groups for continuous measures were analyzed by an independent sample *t*-test and the chi-squared test for categorical measures. The *Z* test was used to compare 2 proportions.

## 3. Results

The study included 44 eyes of 50 subjects complaining of epiphora. They were divided into two groups, the epiphora group one (EG1) received eye drops containing combination of antibiotics and steroids (orchadexoline eye drops, each ml contains 5 mg chloramphenicol, 1 mg dexamethasone sodium phosphate, 0.25 mg tetryzoline hydrochloride, 2 mg hydroxypropyl methyl cellulose, 10 mg *α*-tocopherol acetate (vitamin E), and 8 mg macrogol 400).

The second epiphora group (EG2) received preservative-free artificial tears (sodium hyaluronate-, polyethylene-, and propylene glycol-based) three times daily for four weeks.

Both groups were comparable regarding mean age (49 ± 13 vs 53 ± 11 years, *P* value = 0.2) and sex (males were 38.6% vs 31.8%, female were 61.4% vs 68.2%, *P* value = 0.6), respectively, with no statistically significant difference between both groups (Table 1).

Mean duration of epiphora was measured in both groups before treatment (EG1 = 1.476 ± 0.51 months, EG2 = 1.57 ± 0.52 months) with no statistically significant difference (*P* value = 0.536).

**Figure 1:**
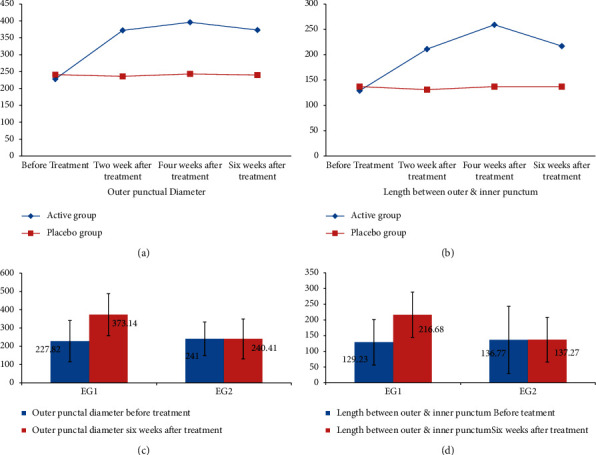
Comparison of outer diameter and the length of the lower punctum between both groups before treatment and during the follow-up period.

Outer diameter and the length of the lower punctum of both groups before treatment and during the follow-up period comparing both groups were assessed objectively by AS-OCT and summarized in Table 2 and Figures 1(a)–1(d).

**Figure 2:**
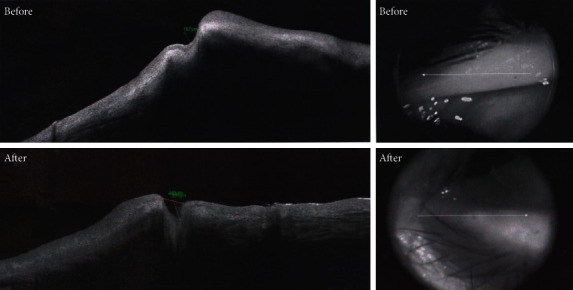
Case 1: OCT punctal changes before and after treatment.

**Figure 3:**
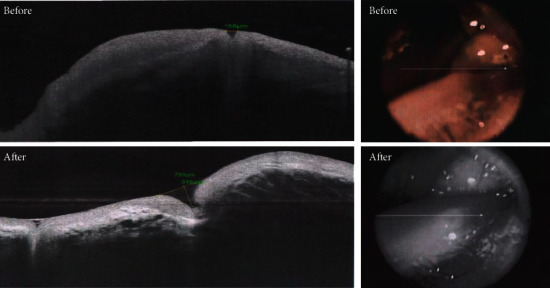
Case 2: OCT punctal changes before and after treatment.

Both groups were comparable regarding outer punctual diameter and length between the puncti before treatment: outer punctal diameters (EG1 228 ± 113 um, EG2 241 ± 115 um, *P* value = 0.5) and length between outer and inner puncti (EG1 129 ± 73 um, EG2 137 ± 72 um, *P* value = 0.6). There was marked improvement of the outer punctual diameter (EG1 373 um ± 92 um, EG2 240 um ± 109 um, *P* value < 0.0002) and length between the puncti (EG1 217 ± 107 um, 136 ± 71 um (*P* value < 0.0008) during the follow-up period. EG1 showed more improvement than EG2 when compared during the follow-up period (Figures 2 and 3).

Subjective assessment of the improvement in both groups using Munk's test and the bitter taste of the orchadexoline eye drops were summarized in (Tables 3 and 4). Both groups showed significant objective improvement using Munk's test, which was more in EG1 when compared to EG2.

## 4. Discussion

The lacrimal punctum is an entry site for drainage of tears to the nasolacrimal duct [14]. Acquired and congenital abnormalities in the size and morphology of the lacrimal punctum and canaliculus may result in excess tears [3]. OCT is an established optical diagnostic technique that was employed for the first time for imaging the anterior segment in 1994 by Izatt [15].

OCT has many advantages, such as being noninvasive, painless, and contactless, with high resolution. Recently, OCT has been used to examine the proximal lacrimal system, and studies have investigated measurement of the anatomical parameters, evaluating the effect of punctoplasty and detecting lacrimal lesions [16, 17].

In this study, we evaluated the role of medical treatment and assessing its effect on resolving epiphora and improving the punctum size by high-resolution AS-OCT imaging comparing punctal parameters between patients and control subjects before and after treatment with topical combined antibiotic and steroid treatment in cases of inflammatory punctual stenosis.

The follow-up period was 6 weeks in both groups of the study. With clinical and OCT follow-up, we found marked improvement of the width and length of the lower puncti in group 1 (*P* value < 0.0001) with more improvement than group 2 when compared during the follow-up period.

In a study of Elalfy et al. [18], they evaluated prospectively the effect of medical treatment (preservative-free hydrocortisone sodium phosphate 3.35 mg/mL eye drops and preservative-free artificial tears based on sodium hyaluronate, polyethylene, and propylene glycol) in the management of inflammatory punctum stenosis guided by spectral domain anterior segment optical coherence tomography (OCT), and they concluded that a combination of preservative-free steroid eye drops and artificial tears causes significant widening of inflamed stenotic punctae and improvement of the epiphora.

We used anterior segment-OCT images for visualizing the lacrimal punctual structural changes as used previously [19] and performed quantitative measurements which help us to monitor disease prognosis and evaluate the efficacy of medical treatment.

The dexamethasone sodium phosphate used in the our study in group 1 exerts its anti‐inflammatory effect in the surrounding conjunctival tissues of the ocular surface by decreasing the release of inflammatory mediators while chloramphenicol as a broad spectrum antibiotic eliminate any underlying subclinical infection.

In a study of Elshorbagy et al. [20], they investigated the role of anterior segment optical coherence tomography in the diagnosis of punctal stenosis and compared punctal parameters before and after medical treatment in the form of preservative-free methylprednisolone 5% eye drops. Their results coincide with our results with improvement in AS-OCT parameters which were useful in monitoring and measuring the efficacy of medical treatment in reducing punctal edema, which results in subsequent reduction in the epiphora symptoms.

As regarding the OCT appearance of obstructed lacrimal puncti, OCT showed the narrow and small opening of the lacrimal punctum with fissure-like lumen of the vertical canaliculus (Figure 2), while in patients diagnosed with advanced punctal obstruction, OCT showed that the opening of the lacrimal punctum was covered by a membrane-like band (Figure 3). These results agree with the study of Hu et al. [21], who investigated the anatomical parameters of normal lacrimal puncta and vertical canaliculus using optical coherence tomography (OCT).

Limitations of this study included the small sample size of cases involved in the research (44 cases) and also, the short period of follow-up was another limitation (six weeks). Further studies are needed in the future with a larger sample size and longer follow-up period.

## 5. Conclusion

Topical combined antibiotic and steroid treatment was an effective method in treating cases of inflammatory punctual stenosis as found by monitoring of punctal parameter changes by AS-OCT. AS-OCT was found to be a useful method for evaluation of the lacrimal punctal parameters especially with different treatment modalities in epiphora cases.

## Figures and Tables

**Table 1:** Demographic data of both groups.

		EG1, *n* = 22		Placebo group, *n* = 22		*P* value
Age	Mean + SD	49 ± 13		53 ± 11		0.2
Sex	Male	17	38.6%	14	31.8%	0.6
	Female	27	61.4%	30	68.2%	0.6

**Table 2:** Outer punctual diameter and length between the outer and inner punctum of both groups during follow-up period of six weeks.

		EG1, *n* = 22			EG2, *n* = 22			*P* value
		Mean	±	SD	Mean	±	SD	
Outer punctual diameter	Before treatment	228 um	±	113	241 um	±	115	0.5
	Two week after treatment	372 um	±	97	236 um	±	109	<0.0001
	Four weeks after treatment	396 um	±	87	243 um	±	110	<0.0001
	Six weeks after treatment	373 um	±	92	240 um	±	109	<0.0002
*P1* value		<0.0001			0.9			
Length between outer and inner punctum	Before treatment	129 um	±	73	137 um	±	72	0.6
	Two weeks after treatment	211 um	±	108	131 um	±	69	<0.0006
	Four weeks after treatment	259 um	±	109	139 um	±	70	<0.0001
	Six weeks after treatment	217 um	±	107	136 um	±	71	<0.0008
*P2* value		<0.0002			0.8			

^*∗*^ *P* ^∗^ *P* value calculates the significant difference of the presented parameters between EG1 and EG2 and was calculated by the independent sample *t*-test. *P1* value calculates the significant difference in the outer punctual diameter in the EG1 and EG2 group before treatment and six weeks after treatment and was calculated by the paired sample *t*-test. *P2* value calculates the significant difference in the length between the outer and inner punctum in the EG1 and EG2 group before treatment and six weeks after treatment and was calculated by the paired sample *t*-test.

**Table 3:** Munk's test results of both groups during follow-up period of six weeks.

Munk's test	EG1 *n* = 22	EG2 *n* = 22	*P* value
	Mean ± SD median (range)		
Before treatment	3.9 ± 1	3 ± 1.3	0.09
	4 (2–5)	3 (1–5)	
Two weeks after treatment	1 + 1	3 ± 1	<0.0001
	2 (0–4)	4 (0–5)	
Four weeks after treatment	1 + 1	3 ± 1	<0.0001
	2 (0–4|)	4 (0–5)	
Six weeks after treatment	1 + 1	3.4 ± 1.6	<0.0003
	1 (0–4)	4 (0–5)	
*P1* value	<0.0002	<0.003	

^∗^ *P* ^*∗*^ *P*value calculates the significant difference of Munk's test between EG1 and EG2 and was calculated by Mann–Whitney *U* test. *P1* value calculates the significant difference of Munk's test in the EG1 and EG2 group before treatment and six weeks after treatment and was calculated by Wilcoxon signed-ranks test.

**Table 4:** Bitter taste results of both groups before and after treatment.

Bitter taste		EG1 *n* = 22		EG2 *n* = 22		*P* value
		Count	%	Count	%	
Before treatment	No	28	63.6	28	63.6	0.8
	Yes	16	36.4	16	36.4	
Six weeks after treatment	No	6	13.6	26	59.1	<0.0009
	Yes	38	86.4	18	40.9	

*PP* value was calculated by Pearson chi-square. No, the patient did not felt bitter taste. Yes, the patient felt bitter taste.

## Data Availability

The data used to support the results of this study are available from the corresponding author upon request.

## References

[1] Hur M. C., Jin S. W., Roh M. S. (2017). Classification of lacrimal punctal stenosis and its related histopathological feature in patients with epiphora. *Korean Journal of Ophthalmology*.

[2] Soiberman U., Kakizaki H., Selva D., Leibovitch I. (2012). Punctal stenosis: definition, diagnosis, and treatment. *Clinical Ophthalmology*.

[3] Soiberman U., Kakizaki H., Selva D., Leibovitch I. (2012). Punctal stenosis: definition, diagnosis, and treatment. *Clinical Ophthalmology*.

[4] Caesar R. H., McNab A. A. (2005). A brief history of punctoplasty: the 3-snip revisited. *Eye*.

[5] Ozgur O. R., Akcay L., Tutas N., Karadag O. (2015). Management of acquired punctal stenosis with perforated punctal plugs. *Saudi Journal of Ophthalmology*.

[6] Fleit H. B., McManus L. M., Mitchell R. N. (2014). Chronic inflammation, pathobiology of human disease. *Pathobiology of Human Disease*.

[7] Kashkouli M., Pakdel F., Kiavash V. (2012). Assessment and management of proximal and incomplete symptomatic obstruction of the lacrimal drainage system. *Middle East African Journal of Ophthalmology*.

[8] Carter K. D., Nelson C. C., Martonyi C. L. (1988). Size variation of the lacrimal punctum in adults. *Ophthalmic Plastic and Reconstructive Surgery*.

[9] Patel S., Wallace I. (2006). Tear meniscus height, lower punctum lacrimale, and the tear lipid layer in normal aging. *Optometry and Vision Science*.

[10] Sung Y., Park J. S., Lew H. (2017). Measurement of lacrimal punctum using spectralis domain anterior optical coherence tomography. *Acta Ophthalmologica*.

[11] Park D. I., Lew H., Lee S. Y. (2012). Tear meniscus measurement in nasolacrimal duct obstruction patients with Fourier-domain optical coherence tomography: novel three-point capture method. *Acta Ophthalmologica*.

[12] Kashkouli M. B., Nilforushan N., Nojomi M., Rezaee R. (2008). External lacrimal punctum grading: reliability and interobserver variation. *European Journal of Ophthalmology*.

[13] Munk P. L., Lin D. T., Morris D. C. (1990). Epiphora: treatment by means of dacryocystoplasty with balloon dilation of the nasolacrimal drainage apparatus. *Radiology*.

[14] Ali M. J., Mishra D. K., Baig F., Lakshman M., Naik M. N. (2015). Punctal stenosis. *Ophthalmic Plastic and Reconstructive Surgery*.

[15] Izatt J. A., Hee M. R., Swanson E. A. (1994). Micrometer-scale resolution imaging of the anterior eye in vivo with optical coherence tomography. *Archives of Ophthalmology*.

[16] Timlin H. M., Keane P. A., Day A. C. (2016). Characterizing the lacrimal punctal region using anterior segment optical coherence tomography. *Acta Ophthalmologica*.

[17] Wawrzynski J. R., Smith J., Sharma A., Saleh G. M. (2014). Optical coherence tomography imaging of the proximal lacrimal system. *Orbit*.

[18] Elalfy H. Y., Elsamkary M. A., Elridy A. M. S., Saad T. M., Rashad S. M., Fawzy S. M. (2020). Medical treatment of inflammatory punctual stenosis monitored by anterior segment optical coherence tomography. *International Journal of Ophthalmology*.

[19] Schmidl D., Schlatter A., Chua J., Tan B., Garhöfer G., Schmetterer L. (2020). Novel approaches for imaging-based diagnosis of ocular surface disease. *Diagnostics*.

[20] Elshorbagy M. S., Shalaby O. E., Eldesouky M. A., Awara A. M. (2020). Anterior segment optical coherence tomography (AS-OCT) guided reversal of edematous punctal occlusion. *Clinical Ophthalmology*.

[21] Hu J., Xiang N., Li G. G. (2021). Imaging and anatomical parameters of the lacrimal punctum and vertical canaliculus using optical coherence tomography. *International Journal of Medical Sciences*.

